# A systems framework for improving clinical trial recruitment: A literature-informed approach

**DOI:** 10.1017/cts.2026.10238

**Published:** 2026-01-21

**Authors:** Blake Zimmerman, Alexandria Moellner, Nasia Safdar

**Affiliations:** 1 Institute for Clinical and Translational Science, University of Wisconsin-Madison, Madison, WI, USA; 2 Department of Medicine, William S Middleton Memorial Veterans’ Hospital, Madison, WI, USA

**Keywords:** Recruitment, community, clinical trials, retention, conceptual framework

## Abstract

**Introduction:**

Recruitment and retention of populations with limited prior participation in clinical trials remains a challenge. Thus, an increased understanding of the complex factors that impede or facilitate recruitment and retention is needed. Adapting the Systems Engineering Initiative for Patient Safety (SEIPS), we developed the Systems Engineering Initiative for Participant-Centric Research (SEIPR) framework that researchers can use to develop, implement, and evaluate interventions to increase trial participation.

**Methods:**

We performed a non-systematic literature review using the digital databases PubMed and Google Scholar to determine factors facilitating and impeding involvement of populations with limited prior participation in clinical trials. From this literature, we developed the SEIPR framework by applying it to the context of recruitment and retention.

**Results:**

We organized key obstacles and evidence-based solutions into five framework components: Person, Tasks and Tools, Technology, Physical Environment, and Organizational Conditions. Common obstacles included lack of awareness of active trials by participants and healthcare providers, patient’s distance from trial centers, lack of access to traditional advertising technology, and mistrust towards investigators, among others. Solutions included promotional strategies appropriate to the regional or social context, decentralizing trials, providing communication technology to participants, partnering with trusted members from the participant’s community and primary care team, using local connections and community centers, financial incentives, and transportation solutions.

**Discussion:**

The SEIPR framework presents a promising tool for investigators interested in increasing participant breadth in clinical trials. Future research is needed to explore real-world applications and assess its effectiveness in recruiting and retaining broad populations.

## Introduction

Clinical trials are essential for advancing innovative medical therapeutics and diagnostics [1]; yet to be effective, their participant population must be representative of the overall population. In the past, clinical trials have often overlooked participants from some demographic groups, including individuals with Tribal nation affiliations and those from rural or less populous urban communities (e.g., African American, Latino, etc.) [2], and their efforts to recruit and retain these groups have shown little improvement in recent years [3,4]. Failing to recruit participants from a broad range of backgrounds can lead to three major consequences: (1) reduced likelihood of meeting accrual goals, (2) limited generalizability of trial outcomes to broader populations, and (3) profound implications for the economy [5]. Therefore, focusing on recruiting and maintaining sufficient research populations from broad backgrounds remains a critical consideration in trial efforts. Increasing participant breadth not only improves the generalizability of clinical trials but also increases enrollment, helping studies meet their accrual goal. Trials that fail to meet these goals face a higher risk of termination [5], wasting valuable time and monetary resources without yielding meaningful scientific insights. When clinical trial outcomes lack generalizability, it worsens patient outcomes and exacerbates existing discrepancies in health among different populations. For example, a study of pulse oximeter devices revealed that patients with darker skin tones were almost three times more likely to have undetected hypoxia compared to patients with lighter skin tones [6], hindering accurate decision-making regarding a patient’s treatment [7]. This factors into populations that have limited prior representation having, on average, shorter lifespans and poorer quality of life compared to traditionally represented populations (e.g., White or lighter-skinned individuals) [8]. These differences in mortality, morbidity, and subsequent loss of work are estimated to cost the United States trillions of dollars over the next few decades [9].

While it is evident that the lack of population breadth in participant backgrounds in clinical trials is problematic, efforts to understand and address the underlying obstacles have not been wholly successful. In response, several conceptual frameworks have been developed to explore these challenges and to provide a process for incorporating cultural considerations into recruitment strategies [10–15]. For instance, the Ford et al. conceptual framework was created to assist in the development of trials to promote participation of populations not typically included in cancer research [10]. This framework includes four major categories: the Barriers/Promoters of “Awareness,” “Opportunity,” and “Acceptance/Refusal,” as well as “Moderators/Sociodemographic Factors,” to help identify factors that impact or influence participation and guide researchers in trial design and planning to improve enrollment [10]. Although useful, the Ford et al. conceptual framework does not address nuanced interactions that can occur between these categories. For example, the framework does not define any interactions between each of the Barriers/Promoters categories, instead placing the emphasis solely on external moderator and sociodemographic factors. Additionally, the framework is specifically aimed at cancer trials and participants without a broader applicable scope.

Similarly, Mainous et al. developed a conceptual framework that sought to improve trial recruitment efforts by directly defining the “Community” as an important piece to consider when looking to improve broader participation in clinical trials [11]. Mainous et al. expanded upon an earlier framework that emphasized the importance of engaging local trusted entities (e.g., religious leaders) in recruitment via the “trust triangle,” consisting of the investigator, the participant, and the participant’s physician [12]. Later, the “trust triangle” was adapted into a “circle of trust” to better align with the cultural needs of their target populations, incorporating community to help recruit American Indian and Alaska Native populations [11]. However, both the “trust triangle” and the “circle of trust” solely focus on interpersonal relationships without expansive consideration for other factors that impact trial recruitment like geography, methods of recruitment, etc [11,12].

Lastly, the Thakur et al. framework provided a multilevel approach to improve trial recruitment [13]. The framework describes levels that grow in scope, starting at the individual level and ending at federal/policy level, providing evidence-based strategies targeting social and structural challenges [13]. However, these strategies are highly specific, sacrificing potential flexibility to be applicable to numerous separate and unique trials. There is also limited interaction between the levels, with scale being the key difference demarcating the individual strategies described within the categories. Additionally, the broadest level of the framework describes grand-scale structural and policy changes, which are important and necessary, but not entirely applicable or practical to individual trial efforts.

While all these frameworks have their utility, they are often limited in scope due to their separate categories, their focus on specific populations, or limited interaction between categories, which decreases their overall usefulness as they risk missing obstacles related to highly interconnected categories or larger populations (Table 1). Consequently, frameworks addressing trial recruitment and retention should be approached from a systems perspective considering all categories as an interwoven system.

**Table 1.** Examples of previous frameworks designed to improve trial accrual

Framework	Key components of the models	Population	Intended use
Ford et al Framework^10^	Study Design Interventions Moderators/Sociodemographic Factors Awareness Barriers/Promoters Opportunity Barriers/Promotors Acceptance/Refusal Barriers/Promoters Awareness Opportunity Acceptance/Refusal Measures of Success	Racial and ethnic minorities, adolescents, older adults, rural residents, and individuals of low socioeconomic status (cancer-related trials).	Provide an evidence base for designing etiologic and intervention research that addresses barriers to participation among populations not typically included in cancer-related trials.
Circle of Trust^11^	Participant Trusted entity Community Investigator	American Indian (AI)/Alaska Natives (AN) participants.	Help investigators and the AI/AN community work together to promote inclusion of AI/AN populations in clinical trials to improve health outcomes.
Triangle of Trust ^12^	Specialist Physician Investigator Minority Serving Referring Physicians Referring Physician’s Patients	Minority patients.	Build trust between specialist physician investigators and minority-serving physicians and ultimately potential participant population.
Thakur et al Framework^13^	Patient Advocacy Community Engagement Individual Interpersonal Systems/Institutional Federal/Policy	Minority patients.	Increase minority participation in trials by addressing multilevel barriers, starting with the foundation of community engagement and patient advocacy, advancing to systemic changes, and ending with tangible recommendations that target barriers.
Systems Engineering Initiative for Participant-Centric Research (SEIPR)	Research Participant Person Technology Tasks and Tools Physical Environment Organizational Conditions	Populations with limited prior participation in clinical trials.	Allow researchers to effectively organize and implement recruitment and retention strategies to increase broad participation in clinical trials.

*Note:* *The language in this table reflects the original language used in the cited publications.

Recognizing the need to better understand the obstacles to clinical trial recruitment and retention, our objective was to adopt a more expansive framework by systematically organizing and addressing these obstacles, and their evidence-based solutions, through a non-systematic review of published literature. Our goal was to support the development, implementation, and evaluation of interventions that enhance involvement from populations with limited prior participation in clinical trials and further improve the recruitment and retention process through our framework.

We adapted the Systems Engineering Initiative for Patient Safety (SEIPS) model, a framework introduced to reduce widespread errors in patient care by recognizing and addressing issues within the healthcare system [16], to apply it to trial accrual. This resulted in the development of the Systems Engineering Initiative for Participant-Centric Research (SEIPR) model. The SEIPR model is designed to allow investigators to think about trial recruitment and retention cohesively, from a systems perspective. By expanding on how these challenges are addressed, we hope that investigators will begin implementing the SEIPR model into trial strategies to help increase broad participation within clinical research.

## Methods

### Non-Systematic literature review

Relevant peer-reviewed publications were identified from a search of PubMed and Google Scholar online databases between November 2023 to April 2024. The focus was to assess the effectiveness of recruitment approaches for populations with limited prior participation in clinical trials, both perceived by participants and reported by the articles’ authors (see “Results”). Keywords to search these databases were selected based on verbiage previously used within scientific literature and included: “clinical trials,” “recruitment,” “retention,” “underrepresented,” “diversity,” “minorities,” “urban,” “rural,” “race,” “racial,” “ethnicity,” “barriers,” “community,” “engagement,” “participant,” “participate,” “research,” “cultural,” and “conceptual framework.” Two authors (BZ and AM) searched for and reviewed the articles. Any issues that arose were resolved through discussion. Articles were included in the review if they were peer-reviewed and published between 2003 and 2024 to reflect the current landscape of recruitment and accrual obstacles and focused on clinical trial recruitment and retention among populations with limited prior participation, particularly those identifying challenges and strategies used to overcome them. Articles were not selected if they did not focus on clinical trials, did not include recruitment and retention strategies or obstacles, or if they lacked relevance to populations with limited prior participation.

### Developing the systems engineering initiative for participant-centric research (SEIPR) framework

The SEIPS framework, which is structured around five components-technology, tasks and tools, person, environment, and organization-to assess patient safety [16], served as the foundation for creating our SEIPR conceptual framework for clinical trial recruitment. We adapted these components by applying new definitions, informed by our non-systematic literature review, that are relevant to trial recruitment and retention (Table 2), and then built connections between the components by examining their relationships to determine how they were interconnected. This new framework allows researchers to effectively organize and implement recruitment and retention strategies to increase broad participation in clinical trials.

**Table 2.** Components within the Systems Engineering Initiative for Participant-Centric Research (SEIPR) model

Person	
Definition:	The individuals who may have either a personal or professional relationship with the potential participant.
*Examples*	
Family. Household Members. Friends. Physicians or other care team personnel. Research study teams. Community Members.	

**Tasks and Tools**	
Definition:	The specific actions, methods, interventions, or items used by investigators to recruit participants.
*Examples*	
Cultural and Linguistic-sensitive promotional materials (e.g. “pamphlets”). Adverts, commercials, promotional community campaigns, etc. Actively developing relationships with community members.	

**Technology**	
Definition:	Specific “Tools” used for trial recruitment that come from a technologically significant source.
*Examples*	
Radio, Television, Internet Advertisements, etc. Cellular Devices. Artificial Intelligence (AI). Electronic Health/Medical Records.	

**Physical Environment**	
Definition:	The physical location and/or living conditions of the potential participant.
*Examples*	
Rural or Urban Environments. Trial Locations Relative to Participant (i.e., Geographical Distance). Travel/Transportation Options Available.	

**Organizational Conditions**	
Definition:	The factors that govern the bureaucratic or structural organization of a clinical trial.
*Examples*	
Number of Trial Locations (e.g., Clinics, Hospitals, etc.). Centralization of Trials (i.e., ratio or ability for “remote” or “virtual” versus “in-person” appointments, check-ups, or interventions). Eligibility Criteria of the Trial.	

## Results

### Developing the systems engineering initiative for participant-centric research (SEIPR) framework

**Figure 1.**
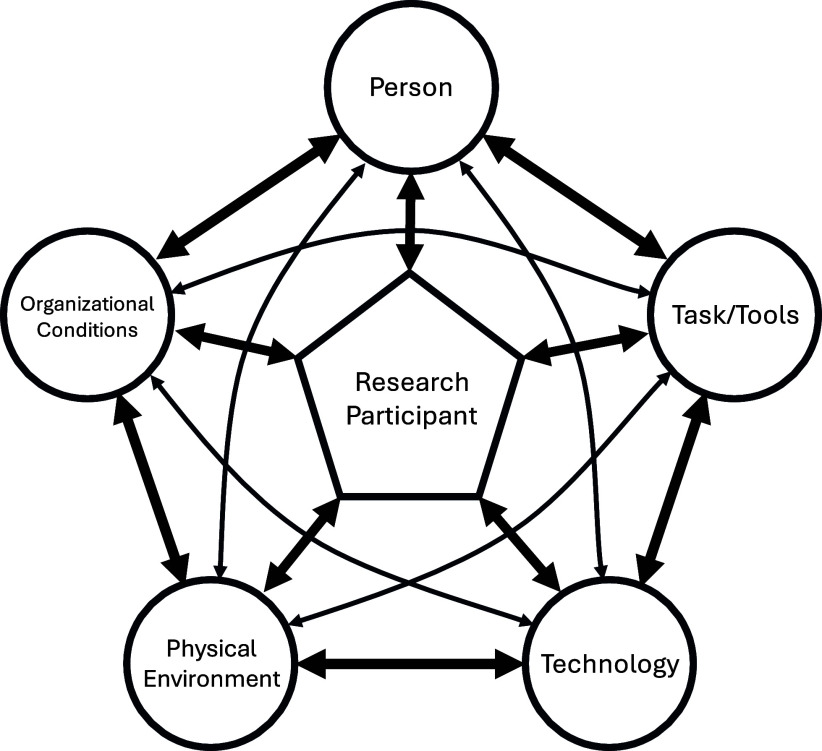
Visual representation of the Systems Engineering Initiative for Participant-Centric Research (SEIPR) framework with the research participant at the center interacting with the key components identified for trial recruitment and retention.

The SEIPR framework defines and contextualizes key factors that contribute to the obstacles of recruiting individuals from populations with limited prior participation in clinical trials. A graphical model (Figure 1) was created to show how these five system components are interlinked, recognizing the participant as an important part of the framework whose personal experiences ultimately impacts recruitment and accrual outcomes. Each component highlights key factors that clarify how to address relevant obstacles and demonstrates how the components are interconnected, helping to maximize efforts to overcome challenges in enrolling populations with limited prior participation in clinical trials.

### The person

In SEIPS, the Person was at the center of the framework. In the SEIPR framework, the importance of interpersonal relationships in trial recruitment has been emphasized. As such, the Person has been formally made into its own category separate from the person who is the research participant. Within SEIPR, the Person can be defined as any individual who has a relationship with the potential participant (personal or professional) and whose opinion about a clinical trial can significantly influence the potential participant’s decision to join [17]. The Person(s) can be a family or household member, friend, physician or other care team member, or member of the participant’s community.

#### Personal relationships

Familial experiences can shape participants’ perceptions and prior assumptions towards clinical trials and the investigators conducting them [18]. Family can be a powerful motivator, and if the potential participant believes participating in a clinical trial can benefit their family members in the future, they are more likely to join [19]. However, if a family member has had a negative experience with a clinical trial during recruitment or participation, such as encountering an inconsiderate or indifferent investigator or seeing no personal or community improvement, the potential participant may be less inclined to participate [19]. When examining how family influences recruitment, it is essential to consider the cultural significance family plays within the population of interest. For example, in rural and Hispanic communities, there is often a strong emphasis on the family unit, with decision-making that prioritizes the well-being of the family over the individual [20–21]. Therefore, recruitment approaches should pay close attention to the relationship between the potential participant and the Person – the family fits into this category in the cases above – when appropriate in order to achieve better trust and improve odds of successful recruitment.

Similar considerations should be made when community plays a central role in the lives of the population of interest, as is often the case in rural and American Indian and Alaska Native populations [11,22]. By engaging trusted community members, investigators can build constructive relationships that foster trust among potential participants and establish reliable sources of information about the clinical trial within the community. Trusted community members can include local religious figureheads, members of local government, and others trusted by the greater community (e.g., elders, prominent community member, etc.) [23]. Investigators should strive to build partnerships [11,15,18,23] so they can ensure the voices and cultural considerations of the community are incorporated in all trial decisions, including the recruitment and retention efforts.

#### Professional relationships

A relationship between the potential participant and the trial representative (e.g., the investigator, primary care physicians, trial coordinators, etc.) is critical for successful recruitment. For example, there is built-in trust between a potential participant and their primary care physician when compared to an unfamiliar trial recruiter [24]. Therefore, involving primary care providers during the recruitment process may alleviate any mistrust that comes with recruiting populations with limited prior participation in clinical trials. If primary care providers cannot be accessed, consider working with representatives from similar demographic backgrounds [25], as trust can be built when a potential participant can connect and communicate in their preferred language or shared culture [20].

### Technology

In the SEIPS framework, Technology was grouped into a Tools and Technology component and was defined as the objects people use to perform tasks or support others in performing tasks. However, given the rise of technology in healthcare and clinical trials, specifically with organization, implementation, and tracking [26], it warrants recognition as a distinct component in the SEIPR framework. Technology ranges from information and communication technologies to physical equipment. Information technology can help investigators identify patients who meet their trial criteria using Electronic Health Records (EHR) and Electronic Medical Records (EMR), even using artificial intelligence (AI) to further enhance their searches, ultimately increasing their recruitment volume [27]. Accessing sensitive patient information through EHRs can, however, introduce regulatory complexities. Another potential use for AI would be to efficiently generate recruitment materials tailored to specific populations, ensuring the content is both clear and culturally relevant [27].

Targeting populations with limited prior participation can have communication challenges, and technology can help with both recruitment and retention. Rural communities that are geographically isolated could be difficult to advertise to through physical means (e.g., a billboard) and may necessitate considering alternative recruitment strategies such as radio and television communications. To maintain close contact with participants with limited resources, clinical trial may provide cellular devices when other conventional methods of communication are unavailable to help with retention [18]. These devices can also be used to track participant progress through mobile applications developed specifically for trial use [18]. However, clinical trials involving cellular devices may face an increased financial burden due to the need for app support, development, or subsidizing device access for participants. Participant hesitancy or unfamiliarity with technology may further hinder recruitment and retention.

### The tasks and tools

In the SEIPS framework, Tasks were treated as a distinct component and defined as the procedures and activities performed by an individual or team, along with their associated description or characteristics. Our SEIPR model combined Tasks and Tools into a broad component and defined it as any specific action, method, intervention, or item used by investigators to recruit individuals from populations with limited prior participation into their trial. They are often direct, easily definable, and used to overcome obstacles that uniquely affect the investigator’s target population.

When recruiting populations, commonly used tools are advertising strategies and promotional materials. Tasks, however, involve broader actions such as building collaborations or partnerships that support trial recruitment and retention efforts. In addition to being an independent component, Tasks and Tools are often connected to other components in the conceptual framework, making them interlinked. For example, the lack of clinical trial awareness by potential participants is a large factor impacting recruitment [15,18,20]. To increase awareness of clinical trial recruitment, investigators can collaborate (e.g., Task and Tool component) with primary care providers (e.g., Person component).

Investigators can also use various advertising strategies, particularly those that can reach a broad audience over long distances (e.g., radio advertisements and TV commercials [interacting with the Technology category]), as well as promotional materials (e.g., flyers, pamphlets, etc.) placed within community centers such as town halls, schools, churches, or recreational centers (e.g., Physical Environment component). These materials should also be adapted to meet the needs of broad populations as they are often omitted due to linguistic differences and variations in customs or practices. For example, communicating the complexities of clinical trials in the population’s primary or preferred language, and choosing images or terminology that are adapted to the local context to avoid overgeneralized assumptions or offense, can help build trust and encourage participation [21]. Investigators can even partner with individuals from the targeted population to ensure the materials are adapted to meet the expectations of the served population and are translated correctly [11,20,21].

### Physical environment

To incorporate the spaces in which people exist and interact with others, our SEIPR model defined the Physical Environment as the locations where potential participants live and exist. As home location and culture influences behavior, location also represents the unique challenges associated with recruiting populations with limited prior participation in clinical trials [11].

Two major challenges are the time commitment required and the physical distance a potential participant must travel, both of which are key factors people consider before committing to participating in a clinical trial [14,17,19,20,28]. For instance, an individual living in a rural community may have a long transit time, which can be problematic for those without access to a vehicle, reliable transportation [28], or sufficient resources, particularly when participation involves late appointments that then require overnight stays. Similarly, an individual living in an urban environment may not have access to a personal vehicle to travel and would have to either walk an unreasonable distance or rely on public transit, which can be unreliable and inconvenient or too expensive [18]. Therefore, individuals have to consider taking extra time off work just to travel to the clinical trial, which can limit participation, especially for multi-visit trials. These challenges can be further compounded by an individual’s need for childcare or a companion, such as someone to accompany them due to an inability to travel alone, an inability to drive themselves, or the need for a trusted family member during medical appointments for cultural or linguistic support [19,20].

These challenges can lead to greater financial costs that many individuals cannot afford or are unwilling to sacrifice for a clinical trial. Investigators can attempt to overcome these challenges by providing financial incentives, such as reimbursement or vouchers for travel fees (e.g., fuel, public fares, lodging) and childcare [18]. Investigators could also consider offering medical transportation, cabs, or ride-share services to ferry participants to and from trial centers. By identifying the Physical Environment prior to beginning recruitment efforts, researchers can proactively consider its interactions between other categories of SEIPR to better prepare, potentially avoiding increased trial expenses or delays.

### Organizational conditions

The SEIPR model defines the Organizational Conditions as the factors that describe how a trial is organized (e.g., trial structure and infrastructure) and how participants are selected (e.g., eligibility criteria). Together, these factors determine the number of individuals that can participate and the populations they come from.

#### Trial structure and infrastructure

An example of an Organization Condition that closely interacts with the Physical Environment component would be having a clinical trial that is centralized (i.e., relying heavily on a single or a few trial centers and locations) which is problematic when recruiting populations with limited prior participation [15]. If a trial were fully decentralized or offered multiple satellite locations participation would become more available to eligible individuals. A decentralized trial eliminates the need for participants to visit a physical site, removing travel-related challenges to recruitment. However, these alternatives come with the added costs of increased staffing, which places a greater administrative burden on trial organizers to recruit, train, and compensate additional personnel.

#### Eligibility criteria

Clinical trials often have strict eligibility criteria that are followed when recruiting the study population. Eligible participants must have qualifying conditions (e.g., diseases or genetic markers) and may be omitted based on logistical factors (e.g., inability to meet study visit requirements), which can be problematic if they are too strict and ultimately hinder the recruitment of populations that have been overlooked in past clinical trials [29,30]. Additionally, physicians and other healthcare professionals serving the target population should be made aware that the trial is actively recruiting participants. They should be informed of the trial’s selection criteria, as well as which patients may be suitable for participation. This can collectively improve trial efforts to recruit participants while promoting a broad trial population.

## Discussion

Our review findings indicate that the components within the SEIPR framework are interwoven, working collectively to identify obstacles to recruiting populations with limited prior participation. By understanding how these components are linked, investigators will be able to coordinate and categorize the obstacles to their trial strategies and find potential evidence-based solutions. For example, travel difficulties for patients from rural communities can arise due to physical distance (e.g., Physical Environment) from a single trial center (e.g., Organizational Conditions), which can limit interest in potential participants. Therefore, investigators can be strategic by partnering with trusted community leaders (e.g., Person) to recruit with radio-based (e.g., Technology), locally relevant advertisements (e.g., Tasks and Tools) that include participation incentives like travel reimbursements (e.g., Physical Environment).

Investigators should also consider how a single strategy can be categorized into multiple components. For instance, educating potential participants about the clinical trial process (the strategy) could be categorized as a Task and Tool if investigators use an informational pamphlet (i.e., a method used for recruitment). Alternatively, it could be categorized as a Person if the investigator decides to partner with primary care providers or community leaders (i.e., individuals who hold an influential relationship with the potential participant). By using a systematic approach for recruitment and retention, clinical trials can increase broad participation by implementing evidence-based solutions.

While improving participant representation in clinical trials has been a major goal of healthcare systems and clinical researchers, current resources are limited in their scope. Using a literature-informed approach, we adapted the SEIPS model (a framework traditionally used to recognize and address challenges within healthcare systems [16]) to better understand the obstacles to, and evidence-based solutions for, recruiting broad populations. Our novel SEIPR framework offers a foundation for future clinical trials to adopt a more integrative approach, recognizing how various components of recruitment and retention may influence potential participants in distinct ways, shaped by their physical environments and cultural contexts. It is our hope that investigators will implement this framework into their recruitment and retention strategies to help populations with limited prior participation enroll in clinical trials, ultimately increasing breadth in clinical trials.

### Implications for practice

**Figure 2.**
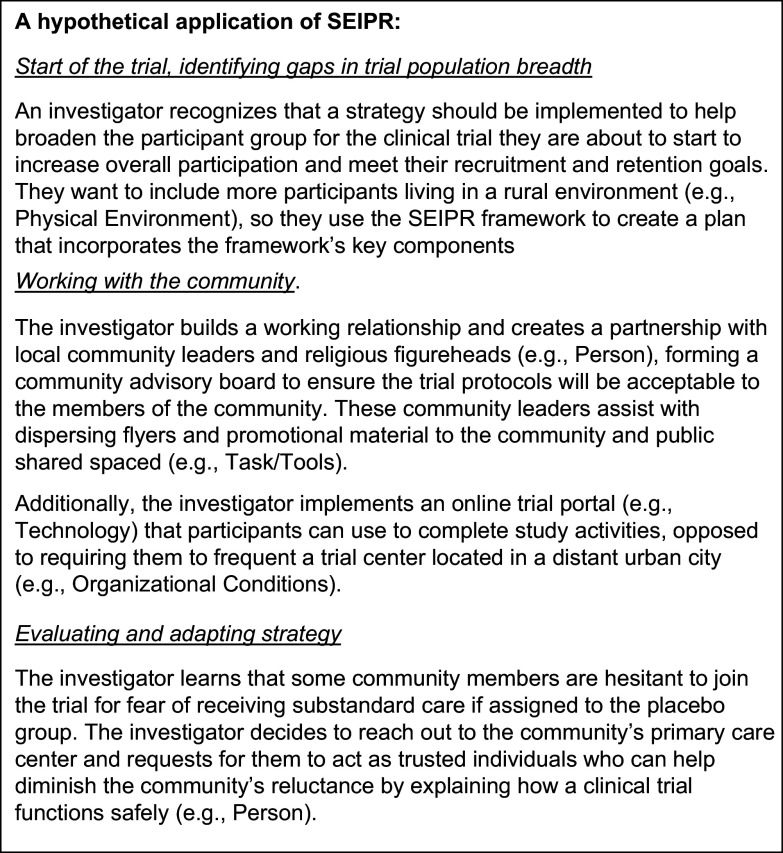
Hypothetical application of the Systems Engineering Initiative for Participant-Centric Research (SEIPR) framework.

The SEIPR framework is a promising tool that helps investigators increase trial participation by tailoring strategies to their target population and research context, all through a systems-based perspective. Its dynamic nature allows investigators to use it during all stages of their clinical trial. During trial development, as investigators start to anticipate their target populations, they can begin identifying potential obstacles and finding evidence-based solutions. Similarly, if unexpected obstacles arise during a clinical trial, an investigator can use the SEIPR model to categorize these obstacles within the appropriate components to understand how they interact with existing strategies. This contextualization can support the development of evidenced-based solutions to move forward effectively. This framework allows investigators to approach recruitment and retention strategies holistically, incorporating multi-faceted methods that reflect the complexity of broad populations and support the overarching goal of increasing population breadth within clinical trials. To view a hypothetical application of the SEIPR framework, see Figure 2.

### Limitations and future research

This paper describes the conceptualization and potential applicability of the SEIPR framework to increase clinical trial involvement of populations with limited prior participation in clinical trials. We employed a literature-based approach to define the framework’s components and identify evidence-based solutions. However, because this approach relied on a non-systematic literature review, some relevant obstacles and potential solutions may not have been captured. While the SEIPR framework offers flexibility, it is not intended to be a comprehensive “catch-all.” It cannot intrinsically define every obstacle or solution researchers may encounter. Rather than serving as a “step-by-step” guide for solving recruitment challenges, SEIPR functions as a blueprint for applying a systems perspective to these issues. Lastly, the SEIPR framework has yet to be implemented and evaluated. Future work should examine its real-world applicability and evaluate the SEIPR framework for recruitment and retention outcomes.

## Conclusion

Recruiting and retaining from populations with limited prior participation remains a key focus for clinical trial investigators. Achieving meaningful representation requires innovative strategies and systems-based approaches that address obstacles to participation. Conceptual frameworks like SEIPR offer an effective way to navigate the ever-growing factors influencing recruitment and retention. By aligning their recruitment and retention goals within the SEIPR framework, investigators will be able to conceptualize and reframe their current and future strategies to maximize participant breadth, produce more generalizable results, and ultimately advance fair health outcomes across all populations.
